# P-918. Rabies encephalitis: Spectrum of MRI findings and predicted probability of abnormality detection based on symptom duration

**DOI:** 10.1093/ofid/ofae631.1109

**Published:** 2025-01-29

**Authors:** Madhav Mohata, Vikas Suri, Paramjeet Singh

**Affiliations:** All India Institute of Medical Sciences, New Delhi, New Delhi, Delhi, India; Post Graduate Institute of Medical Education and Research, Chandigarh, Chandigarh, Chandigarh, India; Post Graduate Institute of Medical Education and Research, Chandigarh, Chandigarh, Chandigarh, India

## Abstract

**Background:**

Rabies is a highly lethal infectious disease and is equivalent to a death sentence for the patient. With currently available tests, the ante-mortem diagnostic sensitivity is only ∼40%. Currently, there are only isolated case reports or small case series exploring the MRI features in rabies encephalitis.

**Figure:**
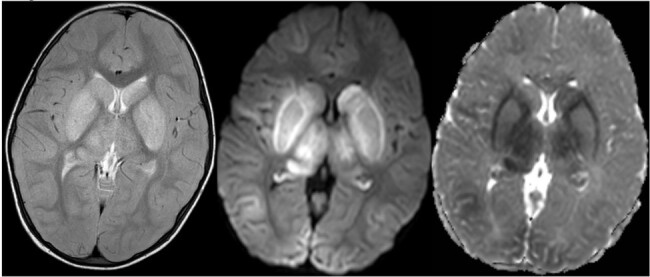

T2 TSE showing hyperintensities in bilateral basal ganglia (caudate nucleus, putamen and globus pallidus) and thalamus and corresponding DWI and ADC images showing true diffusion restriction

**Methods:**

A prospective observational study was designed to explore the patterns of MRI abnormalities in rabies patients. A total of 31 patients were enrolled in the study, out of which 21 underwent Contrast-Enhanced Magnetic Resonance Imaging of Brain and spinal cord.

**Figure:**
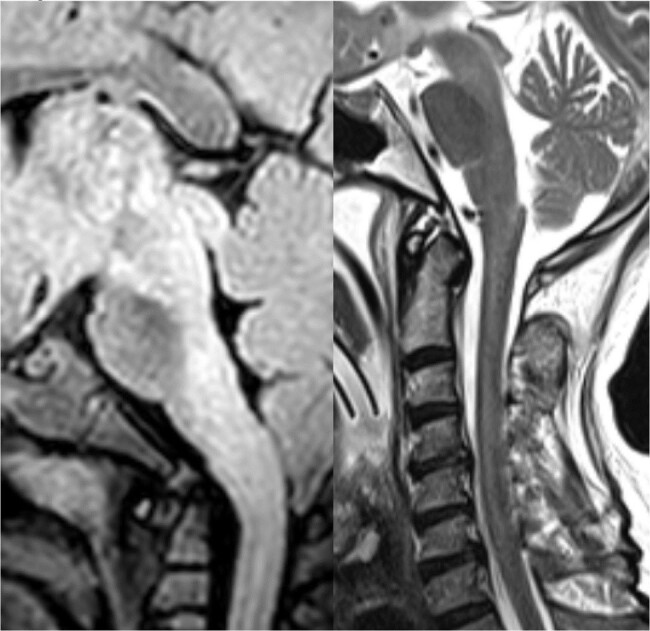

FLAIR/T2 images showing hyperintensities in dorsal brainstem, cervical spinal cord

**Results:**

Overall, MRI was normal in 10(47.6%) patients, whereas 11(52.4%) patients showed abnormalities. Abnormalities were detected in various brain regions with brainstem, spinal cord and basal ganglia being the most commonly affected regions. Other regions such as the cortex, sub-cortical white matter, limbic system, internal capsule, corona radiata, thalamus, and sub-thalamic region were also involved to varying degrees. Diffusion-weighted-imaging showed typical pattern of diffusion restriction in 6 cases (28.5%). No gadolinium contrast enhancement was observed in any patient. Overall, no significant difference in neuroimaging findings were observed between encephalitic and paralytic forms of rabies. In addition, univariate logistic regression analysis revealed that the likelihood of detecting abnormalities on MRI increases with the duration from the symptom onset.

**Figure:**
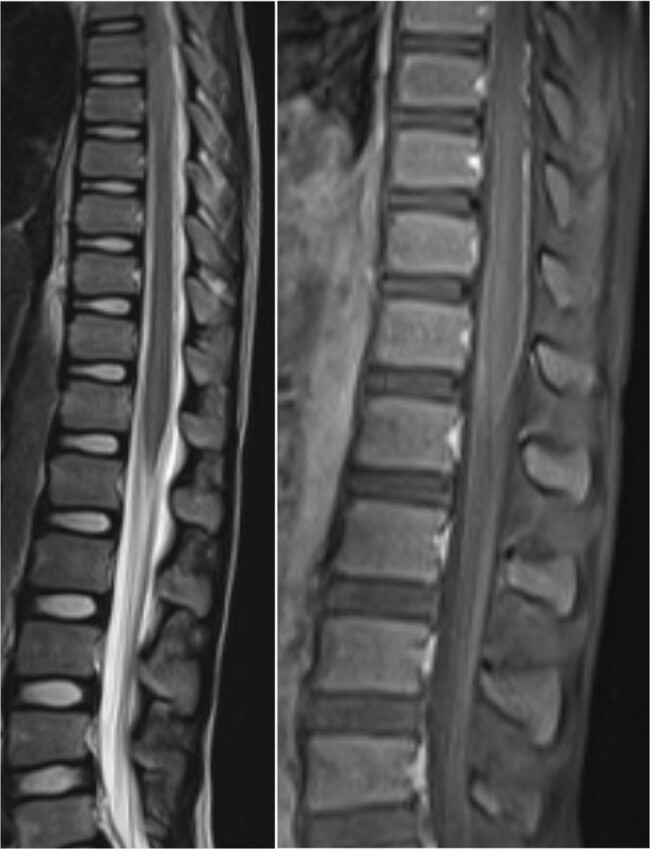

T2 hyperintensity in conus medullaris with contrast enhancing cauda equina nerve roots

**Conclusion:**

Till date this is the largest prospective study (21 cases) exploring the MRI findings in the patients of rabies encephalitis. The study demonstrated certain patterns of brain imaging in rabies which in the appropriate clinical context could significantly aid in establishing the ante-mortem diagnosis of rabies encephalitis. At the same time, the study also highlights the fact that a normal MRI brain does not rule out the diagnosis and that the probability of finding specific abnormalities on MRI increases as the disease progresses.

**Figure:**
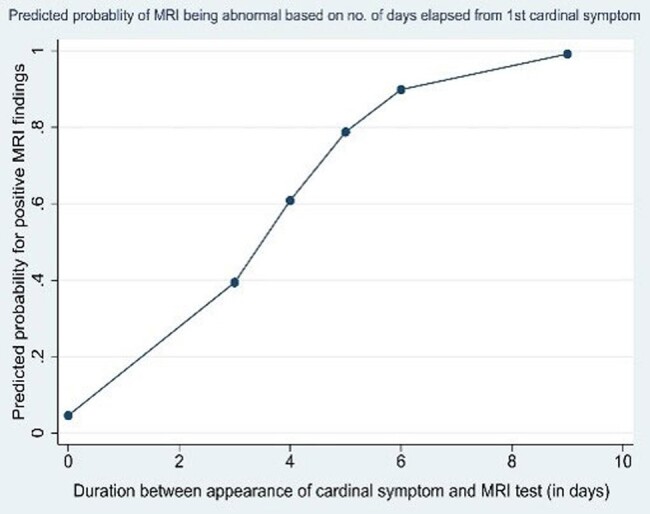

Univariable logistic regression to estimate predicted probability of appearance of MRI abnormality after appearance of 1st cardinal symptom

**Disclosures:**

**All Authors**: No reported disclosures

